# Morphological and Molecular Evaluation of a Gel Based on Hyaluronic Acid and Spermidine for Oral Regenerative Purposes

**DOI:** 10.3390/cells14141047

**Published:** 2025-07-09

**Authors:** Dolaji Henin, Elena Canciani, Daniela Carmagnola, Stefano Ferrero, Gaia Pellegrini, Mariachiara Perrotta, Riccardo Sirello, Claudia Dellavia, Nicoletta Gagliano

**Affiliations:** 1 Department of Biomedical Surgical and Dental Sciences, Università degli Studi di Milano, 20133 Milan, Italy; daniela.carmagnola@unimi.it (D.C.); stefano.ferrero@unimi.it (S.F.); gaia.pellegrini@unimi.it (G.P.); mariachiara.perrotta@unimi.it (M.P.); dottsirello@icloud.com (R.S.); claudia.dellavia@unimi.it (C.D.); 2 Department of Health Sciences, Università del Piemonte Orientale, 28100 Novara, Italy; elena.canciani@uniupo.it; 3 Division of Pathology, Fondazione IRCCS Ca’ Granda Ospedale Maggiore Policlinico, 20122 Milan, Italy; 4 Department of Biomedical Sciences for Health, Università degli Studi di Milano, 20133 Milan, Italy; nicoletta.gagliano@unimi.it

**Keywords:** hyaluronic acid, spermidine, oral wound healing, gingival fibroblasts, organotypic cultures, ECM remodeling

## Abstract

Background: Oral wound healing is a complex process influenced by extracellular matrix (ECM) remodeling and cellular migration. Hyaluronic acid (HA) and spermidine (SP) have shown regenerative potential, but their combined effects on oral tissues remain unexplored. This study aimed to characterize the effect of a gel composed of a mixture of HA and SP on the epithelial and connective compartments of oral tissue separately, evaluating (i) collagen turnover and cell migration on primary human gingival fibroblasts (HGFs) and (ii) epithelial integrity and cell proliferation on gingival organotypic cultures (OCs). Methods: HGFs were cultured, treated with HA-SP gel (1:0.5 HA-SP ratio) and evaluated for collagen types I and III (COL-I, COL-III), matrix metalloproteinase (MMP-1) protein and tissue inhibitor of MMP-1 (TIMP-1) levels secreted by the cells upon gel treatment, compared to CT. HGFs were also analyzed through a wound healing assay. Gingival samples were obtained to set OCs and were treated with different HA-SP formulations (HA 0.2%; 1:0.5 HA-SP ratio; 1:5 HA-SP ratio) to evaluate the beneficial addition of SP to HA for epithelial tissue. OC samples were formalin-fixed and paraffin-embedded and were stained with hematoxylin and eosin and immunostained for Ki-67 analysis. Results: In HGFs, the gel induced increased COL-III gene expression relative to that of COL-I after 48 h and stimulated cell migration, in turn favoring connective tissue remodeling and repair. In OCs, the gel preserved epithelial integrity up to 48 h, with the best effects observed with the 1:0.5 HA-SP ratio. After 72 h, epithelial detachment was more evident in HA formulations, suggesting that SP contributes to epithelial integrity. Conclusions: The HA-SP gel may support oral tissue healing by modulating ECM remodeling and maintaining epithelial integrity. The gel containing HA and SP at the 1:0.5 ratio may provide a promising solution for enhancing wound healing.

## 1. Introduction

Within the oral cavity, the mucosa acts as a physical and microbiological-immune barrier, providing frontline protection against trauma, potential pathogens and exogenous substances [1]. The oral mucosa includes a superficial stratified squamous lining epithelium, containing apposed keratinocytes adhering to one another through cell junctions and laying on a basement membrane between the epithelium and the underlying dense connective tissue. The disruption of the epithelial barrier could happen in cases of surgery, traumas, infections and muco-cutaneous conditions [2]. Alterations in the mucosal structure could lead to local bacterial super-infection, pain, discomfort for the patient and even systemic consequences, especially in conditions in which wound healing is impaired such as diabetes, chronic steroid administration, smoking and others.

Many agents have been investigated for the promotion of oral wound healing, and their use is now commonly recommended in clinical practice. Among these, hyaluronic acid (HA) is a promising candidate due to its structural and regenerative properties, especially when combined with other molecules. HA is one of the essential components of the extracellular matrix (ECM) and contributes to the mechanical properties of the tissue [3]. Beyond its structural role, HA has been widely employed in HA-based gels to promote oral healing and regeneration in the periodontal field. In previous studies, our research group investigated the topical effects of HA on oral wound healing in vitro and in vivo. HA has been shown to enhance ECM remodeling and collagen maturation in the early phases [4], as well as to promote microvascular density and collagen fiber organization [5].

On the other hand, polyamines have been implicated in numerous biological processes, including inflammation, the modulation of oxidative stress and carcinogenesis. At the cellular level, these molecules play a role in growth, differentiation and homeostasis by binding both RNA and DNA. Spermidine (SP) is a low-molecular-weight natural polyamine ubiquitarian in eukaryotic cells, especially in tissues with high cell turnover [6]. SP elicits the inhibition of pro-inflammatory mediator synthesis, including nitric oxide, prostaglandin E2 and cytokines such as tumor necrosis factor α and interleukin 1β. Several in vitro studies have reported that it is strongly involved in inflammatory process modulation, the suppression of pro-inflammatory cytokine synthesis and polymorphonuclear leukocyte chemotaxis regulation [6,7]. Furthermore, SP can promote physiological events, including the induction of autophagy and apoptosis [8,9], immunomodulation, the activation of ECM turnover and mesenchymal stem cell proliferation [8]. Moreover, SP demonstrated a protective action against DNA damage [10]. In a split-mouth randomized controlled clinical trial, Carmagnola et al. (2019) [11], in a periodontal study, reported a significative reduction in probing pocket depth in patients treated with SP gel as an adjunct to non-surgical therapy. This improvement, observed 3 to 6 months after treatment, suggests that SP may enhance and prolong the tissue repair process [11]. Murina et al. [12,13] highlighted the beneficial topical application of a gel combining SP and HA regarding symptoms in patients affected by vestibulodynia [12,13]. A similar formulation combining SP and HA has also been evaluated in dentistry. In a clinical study by Iorio-Siciliano et al. [14], the use of this gel improved the healing of peri-implant soft tissues and led to a larger frequency of resolution in peri-implant mucositis compared to the control group. However, the potential effects and the biological properties of such a combination on oral wound healing in other clinical contexts, such as oral surgery and oral medicine, have not yet been explored. Before its broader application, it is crucial to understand the biological effects of gel containing both HA and SP.

Indeed, although the role of HA in promoting oral healing and restoring the mucous barrier is well known [4,5], the adjunctive effects of SP in this context remain unexplored. This combination may offer a promising approach to enhance oral healing and barrier restoration.

For this purpose, the present study aimed to characterize the biological effect of a gel composed of a mixture of HA and SP on the epithelial and connective compartments of oral tissue separately. The experimental model adopted a molecular and morphological approach and included (i) an in vitro evaluation on primary human gingival fibroblasts to assess collagen turnover pathways and cell migration and (ii) an ex vivo assessment using gingival organotypic cultures to evaluate cellular proliferation and epithelial integrity.

## 2. Materials and Methods

This study followed a two-phase experimental approach to evaluate the effects of a gel combining hyaluronic acid (HA) and spermidine (SP) on the key components of gingival tissue. In the first phase, primary gingival fibroblast cultures were treated with a single gel concentration selected based on the previous literature [11,12], allowing for the assessment of its impact on connective tissue, particularly ECM remodeling and fibroblast behavior. In the second phase, organotypic cultures (OCs) were used to characterize the epithelial response, as they better preserve the three-dimensional architecture of the gingival mucosa. Different SP concentrations in the gel were tested in OCs to determine their specific role in epithelial homeostasis and proliferation.

### 2.1. Sample Collection

The present study was conducted following approval by the Ethical Committee of Fondazione IRCCS Ca’ Granda Ospedale Maggiore Policlinico di Milano (Number 3938, ID 77467, Emendamento Sostanziale n. 1, utilizzo di colture organotipiche, date 16 November 2018, Fondazione IRCCS Ca’ Granda Ospedale Maggiore Policlinico di Milano). All patients read and signed the informed consent form. Fragments of healthy free and attached gingiva were collected using a 15C blade through an intra-sulcular incision, followed by tissue detachment and partial-thickness flap elevation, to obtain biopsies including both the connective and the epithelial layers. The gingival fragments were used to obtain primary cell cultures of human gingival fibroblasts (HGFs) and to set up gingival organotypic cultures (OCs).

Gingival samples were collected at the Maxillofacial Department of the Fondazione IRCCS Ca’ Granda Ospedale Maggiore Policlinico di Milano. The samples were obtained from patients affected by oral carcinoma who required the removal of half of the dental arch. The tissue fragments used in this study were collected from the healthy gingival tissue margin, as confirmed by a pathologist.

The donor patients were recruited according to the following inclusion criteria:

- No preventive radiotherapy or chemotherapy having been undergone;
- No smoking;
- No systemic disease;
- An age of 18–65 years.

### 2.2. Primary Gingival Fibroblast Cell Cultures

Primary gingival fibroblasts were obtained after the collagenase digestion of one gingival fragment. The fragment was carefully washed in sterile PBS, fragmented using a sterile scalpel and incubated in a solution of DMEM (Dulbecco’s Modified Eagle Medium) and 4 mg/mL collagenase I (Millipore, Burlington, MA, USA). The cell suspension was filtered and plated in a T25 flask. The cells were cultured in DMEM containing 2 mM glutamine, antibiotics (100 U/mL penicillin, 0.1 mg/mL streptomycin) and 0.25 μg/mL amphotericin B (Euroclone, Pero, Milan, Italy) at 37 °C in a humidified atmosphere containing 5% CO_2_. The cells were subcultured in T75 flasks, and cell viability was determined by Trypan blue staining.

Gingival fibroblasts were cultured in cell culture medium containing HA 0.2% and 0.1% SP (gel group) or left untreated (CT). HA and SP were kindly provided by Tixupharma (Tixupharma Srl, Milan, Italy). The gel was obtained dissolving in sterile bidistilled water and mixing the two components, HA and SP, which were then diluted in the cell culture medium at the described concentrations (Table 1). The cultures were maintained at 37 °C in an incubator with 5% CO_2_. For molecular evaluations, confluent fibroblasts were cultured in duplicate in 6-well multi-well plates and analyzed at the fifth passage, adding ascorbic acid (200 μM) to DMEM to preserve collagen synthesis, and harvested after 48 h.

### 2.3. Organotypic Cultures

To obtain OCs, four gingival samples were collected and harvested as square-shaped 4 × 4 × 1 mm fragments, immediately preserved in culture medium (HAM F12 supplemented with 10% fetal calf serum, 100 U/mL of penicillin, 100 μg/mL of streptomycin, 2.5 μg/mL of amphotericin B, 100 µg/mL kanamycin) and cultured within 3–4 h at the Thin Section Lab (Università degli Studi di Milano, Milan, Italy) to be processed. The fresh samples were cut in 300–500 μm slices in PBS using a Vibratome VT1200 Leica Microsystem at a low speed to prevent tissue damage (0.01–0.08 mm/s) with 2.95–3 mm oscillations. From each sample, 8 slices were obtained, for a total of 32 slices. The sections were laid on organotypic culture support made of Teflon with 0,4 µm pores (Millicell PICM0RG50 Millipore) and inserted in a well of a 6-well plate filled with 3 mL of culture medium [15]. An extra 1 mL of medium was added to the surface of the tissue to prevent the tissue from dehydration. Gingival tissue fragments immediately processed for light microscopy analysis served as a baseline (B) to compare tissue morphology of untreated (CT) and treated OC samples.

OCs were treated with the gel, diluted in the cell culture medium and containing 0.2% HA alone or in combination with SP at different ratios (Tixupharma Srl, Milan, Italy), at the following working concentrations:

- Group A: HA (0.2%);
- Group B: 1 HA (0.2%):0.5 SP (0.1%);
- Group C: 1 HA (0.2%):5 SP (1%).

Untreated samples served as controls (CT).

As for the in vitro analysis on fibroblasts, the gel was obtained mixing the two components, HA and SP, and then it was diluted in the cell culture medium at the described concentrations.

OCs were maintained at 37 °C in an incubator with 5% CO_2_. For the analysis, OCs were harvested after 24 h, 48 h and 72 h. At each time point, the culture medium was changed, and two slices of tissue were collected for histological and immunohistochemical analysis.

### 2.4. Slot Blot

Slot blot analysis was performed on serum-free cell supernatants of cultured gingival fibroblasts to assay collagen types I and III (COL-I, COL-III), matrix metalloproteinase (MMP)-1 protein and tissue inhibitor of MMP-1 (TIMP-1) levels secreted by the cells upon gel treatment, compared to CT. Briefly, the total protein concentration of cell culture supernatants was assessed using a colorimetric assay (DC Protein Assay, Bio Rad, Segrate, Milan, Italy) and 100 μg of total proteins were loaded on a nitrocellulose membrane, as previously described [16]. After blocking, membranes were air-dried and incubated for 1 h at room temperature with primary polyclonal antibodies to COL-I (1:1000 in 1X Tris-Buffered Saline plus 0.1% Tween 20, TBST) (Abcam ab34710), COL-III (1:1000 in TBST) (Rockland 009-001-105) or MMP-1 (1:1000 in TBST) (Invitrogen PA5-27210). After 1 h incubation with an HRP-conjugated antibody (1:20,000 in TBST), immunoreactive bands were revealed by the Amplified Opti-4CN substrate (Amplified Opti-4CN, Bio Rad, Segrate, Milan, Italy) and quantified by densitometric scanning (UVBand, Eppendorf, Milan, Italy).

### 2.5. Wound Healing Assay

Cell migration in CT and gel-treated gingival fibroblasts were analyzed by wound healing assay in 6-well multi-well plates [17]. The “scratch” was created in confluent cells using a p200 pipet tip. After cell debris removal by DMEM washing, the multi-well plates were incubated at 37 °C in a serum-free cell culture medium and observed under an inverted microscope at different time points. Digital images were captured by a digital camera after 0 and 24 h, and the size of the “scratch” was measured at 0 and 24 h and expressed in pixels. To obtain the migration potential, the wound closure 24 h after the scratch was expressed as a % of the area at 0 h.

### 2.6. Histological Analysis and Immunohistochemistry

Harvested basal (B) and OC samples were formalin-fixed and paraffin-embedded, and 5 µm sections were obtained from each sample. The sections were stained with hematoxylin and eosin to evaluate tissue structure or incubated with a Ki-67 antibody (clone 30-9; Ventana et al. Group, Tucson, AZ, USA), a marker of cells undergoing proliferation [18].

Histological slides were acquired using the Hamamatsu NanoZoomer Series S60 digital scanner (Hamamatsu, Japan) and exported with the NDP.View 2 software (U12388-01) at 40× magnification (ISO-10993-6:2016 [19]).

Tissue morphology and structure were evaluated at each time point by analyzing the architecture of the gingival lining epithelium, the underlying connective tissue and intercellular adhesion. Cell proliferation was assessed through a semi-quantitative analysis of Ki-67 immunostaining, performed on digitized images by quantifying the brown staining pixels by two calibrated operators (DH and EC) [20].

### 2.7. Statistical Analysis

Statistical analysis was performed by the GraphPad Prism v 9.3 software (GraphPad Software Inc.). Data are expressed as mean ± standard deviation (SD). Comparisons of groups were performed using *t*-tests. Differences associated with *p* values lower than 0.05 were considered significant.

## 3. Results

### 3.1. In Vitro Analysis: Gingival Connective Tissue Extracellular Matrix Remodeling

**Figure 1:**
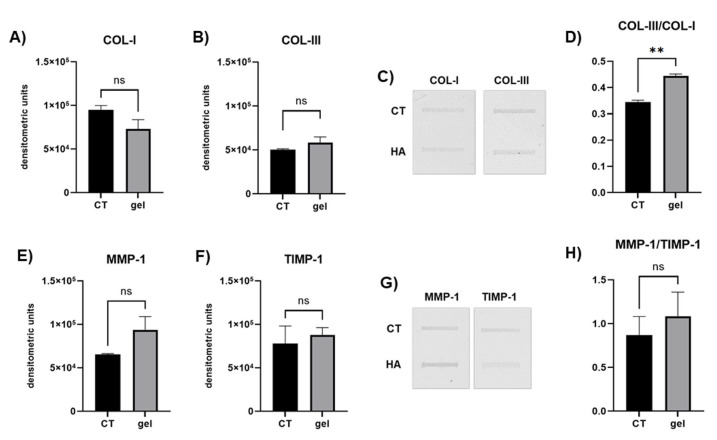
Representative bar graphs showing COL-I (**A**) and COL-III (**B**) protein levels secreted by cultured fibroblasts in the cell culture supernatant. (**C**) Representative slot blot showing COL-I and COL-III immunoreactive bands in untreated (CT) and gel-treated fibroblast cell culture supernatants. (**D**) Bar graph showing the COL-III/COL-I ratio after the densitometric scanning of immunoreactive bands obtained with slot blot analysis. Representative bar graphs showing MMP-1 (**E**) and TIMP-1 (**F**) protein levels secreted by cultured fibroblasts in the cell culture supernatant. (**G**) Representative slot blot showing MMP-1 and TIMP-1 immunoreactive bands in untreated (CT) and gel-treated fibroblast cell culture supernatants. (**H**) Bar graph showing the MMP-1/TIMP-1 balance after the densitometric scanning of immunoreactive bands. Data are expressed asmean ± SD. ** *p* < 0.01.

The analysis of COL-I secreted by primary gingival fibroblasts in cell culture medium revealed that the secretion of the main interstitial collagen of the gingival connective tissue was not influenced by gel treatment (Figure 1A,C). Also, for COL-III expression, CT and gel-treated fibroblasts did not reveal evident differences (Figure 1B,C). However, the COL-III/COL-I ratio significantly increased in gel-treated fibroblasts compared to that in CT (Figure 1D) (*p* < 0.01), pointing to a relatively higher content of COL-III in the gel-treated cells.

To assess collagen degradation pathways, MMP-1 and TIMP-1 expressions were assayed. MMP-1 and TIMP-1 protein levels secreted by gingival fibroblasts in the cell culture medium were not significantly affected by gel treatment (Figure 1E–G). A similar pattern was also observed for the MMP-1/TIMP-1 ratio in both experimental conditions (Figure 1H).

### 3.2. In Vitro Analysis: Cell Migration

**Figure 2:**
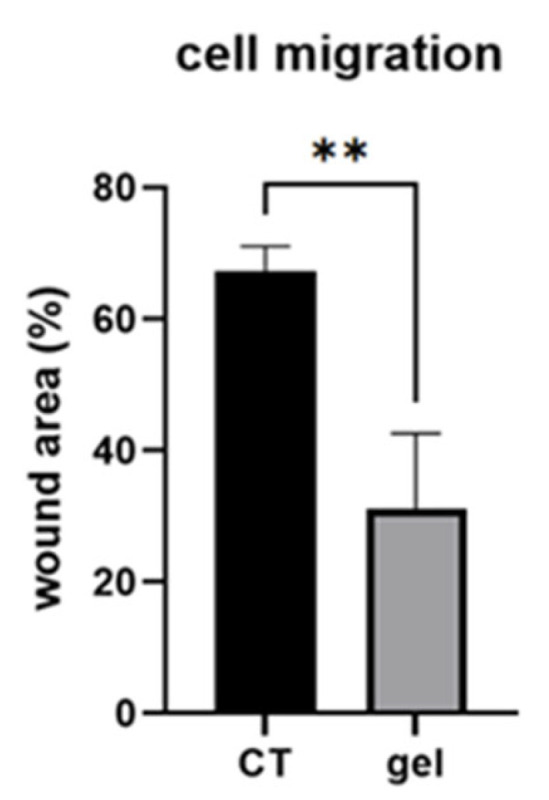
Representative bar graph showing cell migration of CT and gel-treated fibroblasts expressed as %. Data are expressed as mean ± SD. ** *p* < 0.01.

Gingival fibroblast migration was analyzed using the wound healing assay in CT and gel-treated fibroblasts [17]. The wound area at 24 h after the scratch, expressed as a percentage relative to T0, was significantly reduced in gel-treated fibroblasts compared to CT (*p* < 0.01) (Figure 2).

### 3.3. Morphological Analysis of Basal Samples and Untreated OCs and Effect of Gel

**Figure 3:**
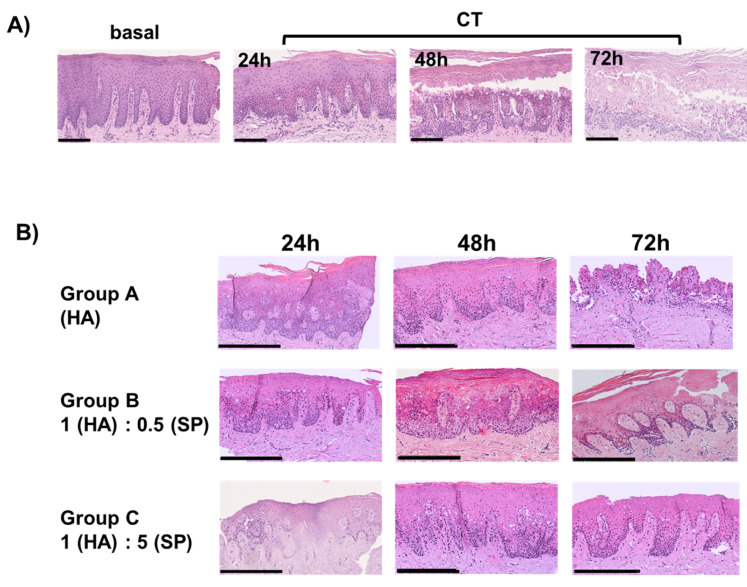
(**A**) Micrographs showing the structure of basal and untreated (CT) gingival OC sections harvested after 24 h, 48 h and 72 h stained with hematoxylin and eosin. Scale bar: 150 µm. (**B**) Representative micrographs showing the structure of gingival OCs sections harvested after 24 h, 48 h and 72 h stained with hematoxylin and eosin. Scale bar: 250 µm.

Gingival tissue fragments immediately processed for light microscopy served as a baseline to compare the tissue morphology of CT and treated OC samples. Light microscopy analysis of hematoxylin and eosin-stained sections of B showed that the tissue structure was preserved, exhibiting the typical structure of the stratified squamous epithelium lining the gingiva and the underlying connective tissue. All the layers of the lining epithelium were clearly represented, and no signs of inflammation, necrosis or dysplastic changes were evident, confirming that the collected tissue was well preserved to serve as a baseline to compare OC samples (Figure 3A). In untreated OCs cultured for 24 h, similarly to that of B, the overall structure of the sample was preserved. Only slight changes were visible in the lining epithelium where the superficial layers tended to detach from the underlying stratum (Figure 3A).

In untreated OCs cultured for 48 h, an extended detachment of the suprabasal layers of the epithelium was detected. These modifications remained evident in untreated OCs cultured for 72 h (Figure 3A). Indeed, at this time point the tissue was characterized by the complete destruction of the lining epithelium. However, at all the considered time points, the gingival connective tissue did not show signs of changes or alterations.

To characterize the effect of the gel on gingival tissue, OC samples of groups A, B and C harvested after 24 h, 48 h and 72 h were analyzed using a light microscope after hematoxylin and eosin staining and compared to B and untreated OC samples (CT).

Morphological analysis of the OCs of group A (HA treatment) showed that the structure and integrity of the lining epithelium were preserved and similar to those of B up to 48 h, while at 72 h the detachment of the suprabasal layers was evident (Figure 3B).

Light microscopy analysis of the OCs of group B (1 HA:0.5 SP) showed that the lining epithelium structure and integrity were preserved after 24 and 48 h (Figure 3B), while at 72 h the detachment of the suprabasal layers and the loss of epithelial integrity became evident (Figure 3B).

In group C (1 HA:5 SP), the effect of the gel was similar to that observed in group B, but the detachment of the suprabasal layers at 72 h was less evident (Figure 3B).

### 3.4. Ki-67 Expression

**Figure 4:**
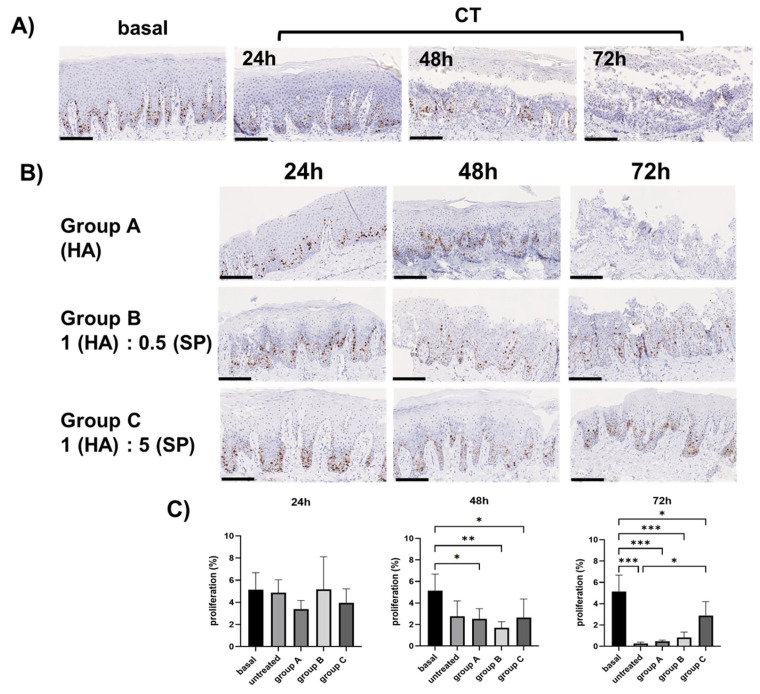
(**A**) Representative micrographs showing Ki-67 immunoreactivity in basal and untreated (CT) OCs harvested after 24, 48 and 72 h. Immunoreactive cells were in the basal layer of the gingival lining epithelium. Scale bar: 150 µm. (**B**) Representative micrographs showing Ki-67 immunoreactivity in the different gel-treated OCs experimental groups harvested after 24, 48 and 72 h. Immunoreactive cells were in the basal layer of the gingival lining epithelium. Scale bar: 150 µm. (**C**) Representative bar graphs showing cell proliferation in the gingival epithelium, expressed as a %, in basal (B) and untreated (CT) OCs and gel-treated OCs of groups A, B and C at the indicated time points. Data are means ± SD. * *p* < 0.05; ** *p* < 0.01; *** *p* < 0.001.

In both basal and untreated OCs harvested after 24, 48 and 72 h, cell proliferation was assayed using an anti-Ki-67 antibody. Light microscopy analysis of Ki-67 immunoreactivity revealed that Ki-67 was expressed in the basal layer of the basal samples, CT (Figure 4A) and treated OCs at all the considered time points (Figure 4B).

The quantitative analysis of Ki-67 expression did not reveal any statistically significant differences in the basal and untreated (CT) samples and the OCs of the different experimental groups after 24 h (Figure 4C). After 48 h, a significant decrease in cell proliferation was detected in groups A, B and C, compared to that in the basal samples (*p* < 0.005). However, the proliferation rate of the treated OCs at this time point was similar to that of CT. After 72 h, the decrease in the cell proliferation of OCs compared to that of the basal samples became more evident, but a significant increase in group C compared to that in the untreated group (CT) was detected (*p* < 0.05).

## 4. Discussion

In vitro studies using 2D cell cultures enable the study of basic cell biology mechanisms, including drug interactions, and have been widely used pharmaceutical research [21]. Organotypic cultures (OCs) mimic the 3D architecture and the natural microenvironment of living tissues [22,23,24,25,26,27,28]. OCs derived from oral and gingival tissues retain the structure of the oral mucosa, possessing a superficial epithelial barrier that can limit and protect the underlying layers from insults [29], and were previously characterized revealing tissue-specific epithelial characteristics [30]. Compared to available 3D systems, OCs better preserve the three-dimensional architecture of native tissue and maintain patient-specific characteristics, making them particularly useful for investigating tissue responses to drugs, molecules and biomaterials [31].

In this study, to evaluate the response of oral tissue to a gel containing HA and SP, two experimental approaches were combined: using primary fibroblast cell cultures and using OCs to investigate the gel’s effect on the connective compartment and the epithelial tissue, respectively.

To explore the effect of HA-SP on the gingival connective tissue, collagen turnover pathways were finely studied at the molecular level in primary human gingival fibroblasts (HGFs), which are the main cells responsible for maintaining tissue homeostasis by secreting the ECM components and the proteases involved in their degradation.

COL-I is the main component of the ECM of the dense irregular connective tissue of the gingiva and oral mucosa [32,33]. COL-I and COL-III levels secreted by gingival fibroblasts in the cell culture supernatants were unaffected by gel administration. Interestingly, however, the COL-III/COL-I ratio was significantly increased in gel-treated fibroblasts, suggesting that COL-III was secreted to a greater extent in the early hours after gel treatment, compared to COL-I. This finding is consistent with the hypothesis that the gel could be effective in favoring gingival healing since during the initial stages of tissue remodeling, the expression of COL-III increases as it is more hydrophilic than COL-I, and this promotes ECM remodeling in the early phases of tissue repair [34].

Collagen content in tissues is the result of a finely tuned dynamic balance between its synthesis and degradation driven by MMPs, especially MMP-1, which is the main collagenolytic enzyme involved in transforming the interstitial collagen triple helix into the two 3/4- and 1/4-collagen degradation fragments [35,36,37].

MMP activation and activity are regulated by TIMPs; among them, TIMP-1, the main inhibitor of MMP-1, binds MMP-1, inhibiting both its activation and activity. The key role of MMP-1 and TIMP-1 played by fibroblasts in gingival connective tissue was previously demonstrated by our group in different conditions, including gingival overgrowth and repair [4,37,38,39,40,41,42].

Our results show that MMP-1, TIMP-1 and the MMP-1/TIMP-1 ratio were not significantly affected by the gel in both experimental conditions, suggesting that the gel did not target MMP-1 balance. According to this finding, we can hypothesize that the HA and SP contained in the gel could affect collagen turnover pathways involving HGFs at the level of collagen synthesis but not at the level of its degradation.

Cell migration plays a key role in maintaining connective tissue homeostasis and favoring tissue healing, also in the gingival tissue. During repair, gingival fibroblasts migrate to the wound site and synthesize cytokines and ECM components to orchestrate the wound-healing process in response to injury or disease and during tissue repair [43,44]. Our results show that HGF migration was significantly induced by gel administration, compared to that in CT. This finding strengthens our hypothesis that the gel could be effective in favoring tissue repair and regeneration by exploiting this effect on cell motility, to favor ECM remodeling to restore tissue homeostasis.

To assess the effect on lining epithelium integrity and proliferation, we tested HA associated with SP in OCs. Previous studies described the beneficial use of HA in various fields of medicine, including dentistry [45,46], revealing that HA seemed effective in favoring periodontal healing after surgical procedures [47]. The effects of SP on oral tissues previously investigated in a periodontal model showed that its association with non-surgical periodontal treatment led to a significant reduction in probing pocket depth after 3 to 6 months, suggesting that spermidine may contribute to long-term periodontal health [11]. However, SP’s role in oral tissue healing and regeneration remains still unexplored.

In this study, the effect of the gel on gingival tissue integrity was assessed in OCs treated with the same concentration of HA and SP used for fibroblasts (group B), or with a gel containing a different dose of SP (group C) or containing only HA (group A). To better describe the effects of the gel, basal samples and untreated CT OCs were also analyzed.

Our data show that basal (B) and CT OCs had similar morphology and well-preserved tissue integrity after 24 h in culture. However, in CT OCs, starting at 48 h, the detachment of suprabasal layers of the lining epithelium was detected. Interestingly, no evident modifications of the underlying connective tissue were detectable at all the time points considered.

Light microscopy analysis of gel-treated OCs confirmed the results obtained with CT after 24 h, showing that epithelial structure was preserved in all the considered experimental groups and, interestingly, also retained up to 48 h while the detachment of suprabasal layers was evident only at 72 h. This finding suggests that the gel elicited a protective effect on the epithelial integrity up to 48 h in gel-treated OCs, compared to that in untreated (CT) OCs. However, when comparing tissue integrity in treated OCs, group A showed the most evident epithelial damage compared to groups B and C. These results lead to the hypothesis that SP, which was not present in the gel of group A, could be effective in maintaining epithelial homeostasis. To test this suggestion, since SP could elicit effects on cell proliferation [48], we analyzed Ki-67 expression in OCs.

Ki-67 expression was similar in basal and untreated (CT) OC samples at 24 h and in all the gel-treated OCs. After 48 h and 72 h the decreased proliferation rate of untreated CT samples compared to that of basal samples was more evident, suggesting decreased cell viability in OCs compared to in vivo tissues. However, after 48 h, Ki-67 expression was similar and preserved in CT and gel-treated OCs. This finding is consistent with the results of the morphological analysis, suggesting that at the 48 h time point, epithelial integrity and viability were still preserved. Conversely, after 72 h, a significant up-regulation of Ki-67 in group C was detected, compared to that in CT, likely pointing to increased cell proliferation induced by the administration of a gel with a higher SP %. However, since this result was obtained in OCs that, at this time point, showed an evident loss of tissue integrity and loss of some epithelial suprabasal layers, it cannot be considered a valid and reliable result. We can also highlight that the significant induction of epithelial cell proliferation triggered in group C should be considered with attention since it could be dangerous if the gel is used in repeated treatments over time. However, in the context of oral healing and regeneration, topical gels intended to promote the initial stages of tissue repair should generally be used for short durations, even in patients with impaired healing, where the process may take several weeks. Additionally, the oral cavity per se is a fluid-rich environment, with saliva and crevicular fluid, which would naturally dilute the topical gel, thereby reducing the potential risks associated with prolonged exposure.

Overall, data obtained on OCs show that the gel used in group B, containing HA and SP at the 1 HA:0.5 SP ratio, is likely the most suitable formulation for the aim of this study, as it preserved tissue structure while also supporting cell proliferation. Interestingly, the gel used in group B in OCs is the same as that used to treat gingival fibroblasts.

Considered as a whole, the results of our study obtained in primary gingival fibroblasts and OCs suggest that the gel containing HA and SP at a 1 HA:0.5 SP ratio could be effective in preserving gingival homeostasis. Indeed, the gel was able to stimulate in fibroblasts the mechanisms involved in gingival connective tissue remodeling during repair and healing but was also effective in preserving tissue structure and integrity, as well as epithelial cell proliferation.

The use of a similar gel with spermidine and sodium hyaluronate was previously described in a clinical study to improve the soft tissue healing of implants with peri-implant mucositis [14]. The study analyzed the clinical outcome following treatment with a unique local application of the gel and demonstrated a significant improvement in clinical parameters. Similarly to the work by Carmagnola et al. [11], the study was conducted within a periodontal model. In contrast, the aim of our study was to further investigate the biological effects of this gel on both the epithelial compartment and the connective tissue of the gingiva, thereby expanding its potential applications. To assess its capacity to promote tissue healing, we evaluated cell migration and analyzed collagen turnover as an indicator of extracellular matrix remodeling. Furthermore, to explore the gel’s regenerative potential, we examined epithelial cell proliferation. These experimental approaches enabled us to characterize the effects of the gel at both cellular and tissue levels, providing novel insights into its mechanism of action.

This gel formulation may prove particularly valuable in clinical scenarios that require enhanced soft tissue regeneration, especially in patients with impaired or delayed healing responses, such as individuals with diabetes, elderly patients, immunocompromised individuals or those undergoing corticosteroid therapy.

In these populations, enhanced healing support could reduce the risk of complications and improve overall recovery outcomes, avoiding super-infections potentially due to oral microorganisms. The gel’s application may extend to various surgical scenarios, such as in dental extractions, in which primary healing often is not achievable, or in cases in which the gingival seal is critical to prevent infection and ensure long-term success, such as in implant surgery and procedures involving maxillary bone regeneration. Additionally, it could also be beneficial in managing minor oral traumas or ulcers that, while not requiring sutures, can cause significant discomfort to patients and may benefit from a topical approach to promote healing and symptom relief. Overall, for preserving tissue integrity and homeostasis, the gel represents a promising support in surgical and healing contexts where enhanced regeneration is needed.

Moreover, our gel formulation may overcome some of the limitations of topical products currently used in clinical practice for oral wound healing. Gels containing antimicrobial agents, such as chlorhexidine, can reduce the risk of infection but may also disrupt the natural oral microbiota, which plays a crucial role in mucosal immune homeostasis [49]. In addition, chlorhexidine has been reported to delay wound healing, making its routine use in clinical practice increasingly controversial [50]. Gels based only on HA are beneficial during wound healing due to their anti-inflammatory and hydrating properties [5]. However, the addition of SP may enhance healing in the later phases, as suggested by Carmagnola et al. (2019) [11], by promoting epithelial cell proliferation and supporting tissue remodeling.

Although our data contribute new insights on the effect of HA and SP on gingival tissue, our study has some limitations. Indeed, the OC experimental model, although mimicking the gingival structure, lacks vascularization and has limited metabolic exchanges, making it not fully comparable to an in vivo system. Vascularization plays a crucial role when assessing the effects of bioactive molecules on tissues, as it facilitates the transport of mediators to the target site in response to treatment while also contributing to the metabolism and clearance. The absence of these physiological mechanisms in the OC model may influence the results, highlighting the need for complementary in vivo studies to validate the findings.

Additionally, considering that both HA and SP are hydrosoluble, and given the dynamic environment of the oral cavity, constantly exposed to saliva and mechanical stress, the dilution and displacement of the gel are important factors to consider. Therefore, for future in vivo studies, the incorporation of mucoadhesive excipients [11] or the use of oral-coating gels, commonly recommended in oral medicine to prolong topical drug contact time, should be considered. Moreover, multiple applications will likely be necessary to maintain sufficient bioavailability and therapeutic effects over time.

## 5. Conclusions

HA-SP gel can promote oral tissue healing by modulating ECM remodeling and maintaining epithelial integrity. The 1:0.5 HA-SP ratio may provide a promising solution for enhancing wound healing. Further in vivo studies are needed to confirm clinical applicability.

## Figures and Tables

**Table 1:** Components of the gel and concentration in the gel and in the cell culture medium for the treatment of gingival fibroblasts.

Gel Components	Concentration in the Gel	Concentration in the Medium
Sodium hyaluronate	2%	0.2%
Spermidine HCl	1%	0.1%

## Data Availability

The data that support the findings of this study are available from the corresponding author upon reasonable request.
